# Increasing ecological sustainability using the combinations of technologies to produce power

**DOI:** 10.1016/j.heliyon.2023.e20567

**Published:** 2023-10-06

**Authors:** Weidong Huo, Muhammad Zulfiqar, Shahida Parveen, Muhammad Rizwan Ullah, Ahmed Chand

**Affiliations:** a School of Finance and Trade, Liaoning University, Shenyang, China; b Department of Business Administration, Government College Women University, Faisalabad, Pakistan; c Lyallpur Business School, Government College University, Faisalabad, Pakistan; d School of Economics and Management, North China Electric Power University, Beijing, China

**Keywords:** Environment sustainability, Hybrid energy technologies, Solar photovoltaic system, Circular economy

## Abstract

The study attempts to analyze the impact of hybrid energy systems on environmental sustainability in the context of the circular economy network. The hybrid energy sources follow the principle of circular economy, which aims to reduce raw material use and waste which is very fruitful in promoting environmental sustainability. However, the study uses quarterly data from Pakistan from 2011Q1 to 2020Q4 to accomplish the proposed study objective. The study constructs 5 hybrid systems of energy to analyze the impact and applies Auto Regressive Distributive Lag Model (ARDL) to estimate the results. It is found that electricity generation through solar photovoltaic (SPV), wind turbine (WND), hydroelectricity (HYDE) and nuclear power generation (NPG) play a positive role in increasing environment sustainability. The results explain that SPV, NPG, and HYDE contribute 2.29%, 2.04%, and 0.42% to environmental sustainability, respectively. Hybrid systems of energy (Hybrid 1,2,3,4 and 5) positively impact ecological sustainability, but the intensity of each hybrid system in sustaining the environment is different. Among five hybrid systems, the hybrid-5 (SPV-WND-HYDE-NPG) energy system is more environmentally friendly and has the highest contribution towards environmental sustainability. The study suggests incorporating hybrid energy systems provides the means of transition toward a circular economy, ultimately promoting ecological sustainability. The study proposes to the officials of the Pakistani government and policymakers to initiate effective policies to encourage hybrid energy systems because the significance of hybrid systems ensures a low carbon economy and makes a path towards sustainable economic development.

## 1 Introduction

In remote regions worldwide, access to electricity is often limited or absent, leading to a heavy reliance on small, isolated diesel generators [1]. The operational expenses associated with these generators are considerable due to the high costs of fossil fuels and the challenges of fuel procurement and storage. To address this issue and promote a transition towards a circular economy, renewable energy sources such as solar photovoltaic (SPV), wind turbine (WND), hydroelectricity (HYDE) and nuclear power generation (NPG) offer a viable alternative to complement engine-driven generators for power generation in off-grid areas [2]. The circular economy, guided by the principles of “reduce, reuse and recycle”, prioritizes the efficient utilization of resources within environmental sustainability (ES) regulations. Researchers have demonstrated that hybrid energy systems, combining different technologies for power production, not only supply electricity but also reduce the life cycle impact of isolated power systems in various off-grid settings [3,4]. The growing adoption of hybrid systems worldwide has made renewable energy production economically feasible and cost-effective by harnessing diverse technologies.

Integrating hybrid systems comprising SPV, WND, HYDE and NPG has become feasible due to the economic considerations in isolated electrification zones. These hybrid systems possess the advantage of flexibility, enabling them to adapt to varying conditions [5]. Additionally, combining different renewable energy sources reduces the need for large battery banks. It diminishes diesel consumption, decreasing fossil fuel consumption and CO_2_ emissions (CO_2_E), a significant contributor to the greenhouse effect and climate change [6,7]. Climate change poses a significant global environmental challenge and profoundly impacts energy planning. Non-renewable energy sources have been implicated in environmental degradation, highlighting the urgency to adopt renewable energy sources, including SPV, WND, HYDE and NPG, as remedial measures [8]. Although some renewable sources are already utilized, their full potential must be explored. Utilizing various renewable energy sources collectively for electricity generation is pivotal in mitigating environmental degradation and promoting a circular economy [9]. Addressing the current power sector concerns, such as environmental pollution and inadequate availability in rural areas, requires innovative solutions like hybrid energy systems [10].

A hybrid energy system represents a well-orchestrated combination of diverse renewable energy sources, such as SPV, WND, HYDE and NPG. This intricate infrastructure design ensures a seamless electricity supply to the circular system while adhering to “reduce, reuse and recycle”. The electricity generated from hybrid systems produces minimal to no toxic emissions, making it environmentally friendly [9]. Beyond its environmental benefits, adopting hybrid systems is economically sound and reliable in meeting diverse load demands with minimal investment and operational costs [11]. The convergence of renewable energy technologies in hybrid systems presents a compelling solution to address energy access and environmental concerns, ushering in a greener and more sustainable future for remote communities.

Given the substantial contributions of renewable hybrid energy systems to ES, this study asserts their potential benefits for developing economies like Pakistan, aligning with the principles of the 3R approach. This approach involves the feasible replacement of conventional fossil fuel systems with renewable or cleaner energy alternatives and the reuse and reutilization of renewable energy resources. By investigating the role of diverse energy hybrid systems in environmental protection, the research makes a notable contribution to the scholarly discourse. While existing literature has explored the impact of SPV and WND hybrid systems on ecological quality [[12], [13], [14], [15]], more research should focus on hybrid energy systems comprising SPV, WND, HYDE and NPG in tandem. The present study seeks to address this gap by delving into the configuration and function of such hybrid energy systems and their potential to mitigate the effects of climate change in Pakistan. Hence, the study answers, "How do hybrid energy systems contribute to ES? We, thus, aim analyzing the impact of hybrid energy systems on ES.

The motivation behind this research emanates from the pressing global imperative to confront ES challenges, particularly in energy production and its consequential impact on the environment. The escalating apprehensions regarding climate change, resource depletion and waste generation underscore the urgency for novel approaches to power generation that align with the tenets of the circular economy. To address this critical concern, the study explores the potential of hybrid energy systems in advancing ecological sustainability by synergistically integrating diverse renewable sources, such as SPV, WND, HYDE and NPG, within the circular economy framework. The research endeavors to evaluate their affirmative contributions to ES. By employing quarterly data from Pakistan and applying the Auto Regressive Distributive Lag Model, the study aims to furnish rigorously substantiated insights into the efficacy of various hybrid energy systems. Notably, identifying Hybrid-5 (SPV-WND-HYDE-NPG) as the most ecologically sound option underscores the salience of investigating such amalgamations of technologies to attain ecological sustainability. The research outcomes and ensuing recommendations hold substantial significance for policymakers and the Pakistani government, as they underscore the potential of hybrid energy systems to catalyze a transition towards a low-carbon economy and sustainable economic development. By illuminating the pivotal role of innovative energy solutions in safeguarding the environment, this study contributes to the global endeavor to combat climate change and foster a more sustainable future. The remaining research is structured as follows: Section 2 presents the review of existing literature and hypothesis, section 3 provides the methodology, section 4 elaborates on the empirical results, and section 5 presents the conclusions and implications.

## 2 Literature review

Over the past decade, the world has grappled with a pressing issue of global warming, marked by a rapid surge in carbon emissions, negatively impacting the environmental quality of both developed and developing nations. This deterioration poses a significant threat to sustainable development, leading to severe ecological challenges. Researchers have consistently highlighted that energy derived from conventional sources remains the primary driver of global greenhouse gas (GHG) emissions, contributing to the degradation of the environment [[16], [17], [18]]. Conversely, energy generated from clean and renewable sources has shown promise in enhancing environmental quality [19,20]. This focus on the energy-environment nexus has spurred researchers to publish studies investigating the link between energy production and its environmental impact.

### 2.1 Literature review in international context

Numerous researchers have explored the contributions of energy produced from combining different renewable sources in improving environmental quality. For instance, Gunerhan et al [21]. investigated the effect of solar photovoltaic (SPV) on CO_2_E and compared the results with conventional voltages. They found that SPV had fewer contributions to CO_2_E and more favorable environmental effects than traditional voltages. Tsoutsos et al. [22] examined the influence of SPV on CO_2_E and found a significant impact of SPV on CO_2_E. Gish et al. [23] identified SPV as a boundless source that had a minimal contribution to increasing CO_2_E. Mahmud et al. [24] conducted research and presented a life cycle valuation of an SPV and solar thermal environment. The results of their study revealed that SPV had less significance in increasing CO_2_E than solar thermal.

The study identified that the structure of solar thermal offered more than double (100%) discharges to air compared to SPV, which has only 23.26% discharges. Kommalapati et al. [25] studied the literature on SPV and revealed that SPV had fewer contributions to increasing CO_2_E. While Mancini et al. [26] used ecological footprints as a proxy for environmental degradation and found no significant relationship between SPV and ecological footprints. Some researchers indicated that renewable hybrid systems have a positive role in protecting the environment. This is because of the reason that hybrid systems accumulate renewable energy at a low cost which not only improves the environmental quality but also advances energy efficiency [27]. Lang et al. [28] examined the influence of hybrid systems on CO_2_E and showed that energy consumption was reduced by using a hybrid system that reduced CO_2_E.

The study also indicated that hybrid systems lessened carbon emissions for a single vehicle. Wang and Zhang [29] explored the impact of the wind-generator hybrid system on CO_2_E and compared the outcomes with the conventional system. The results of their study revealed that, as compared to traditional systems, wind generator hybrid systems had fewer contributions in increasing CO_2_E. They suggested diverting attention from conventional sources to hybrid sources to protect the environment. Ashok [30] used the method of environmental risk assessment and revealed that hybrid systems of SPV and wind-generator were environment friendly and had contributed to environmental degradation.

Similarly, Shezan et al. [31] also found the positive environmental effects of hybrid systems. Rehman and El-Amin [32] worked on the relationship between hybrid power systems and CO_2_E. For this purpose, they selected 3 types of hybrid systems and compared their contributions to the environment. They found that SPV had minor contributions to CO_2_E while diesel generators had higher contributions to CO_2_E. Nema et al. [33] investigated the relationship between solar photovoltaic and diesel hybrid systems and CO_2_E. They found insignificant contributions of these hybrid systems in CO_2_E. They concluded that both sources were environmentally friendly and easily obtainable.

Sawle et al. [34] studied the relationship between SPV and wind-battery hybrid systems and their environmental effects. The study concluded that these hybrid systems had a strong positive impact on ecological sustainability and were used as a substitute for each other. The study also found the economic feasibility of these hybrid systems. Elhadidy and Shaahid [35] investigated the influence of wind battery and geothermal hybrid systems on CO_2_E. The study found that both hybrid systems significantly reduced CO_2_E while geothermal had more critical contributions. Nwafor [36] evaluated the emission appearances of diesel generators. For this purpose, he assessed rapeseed methyl ester (RME), liquescent oil for diesel engines, and compared it with fuel to accomplish emission desires. The finding showed a reduction in CO_2_E when the diesel generator was running on RME compared to fuel. Üçtuğ and Azapagic [37] investigated the influence of hybrid systems on CO_2_E and compared it with grid electricity. The study found that as compared to grid electricity, hybrid systems had fewer contributions in increasing CO_2_E. The core macroeconomic theory also supports that the production or consumption of power through two or more combinations of renewable sources not only reduces the dependency on inadequate resources but also protects the environment by reducing the negative externalities from the production process [38].

Shifting the geographical scope to China, Qin et al. [39] empirically explored the impact of green finance and the blockchain market on carbon neutrality. Their findings indicated the positive and negative effects of the blockchain market's influence on carbon neutrality. Additionally, the research shed light on green finance's persistent role in promoting carbon neutrality, albeit at a relatively slower pace with less influential implications than the blockchain market. Su et al. [40] examined the impact of technological innovation on carbon neutrality. Their findings presented a nuanced perspective, revealing a dual effect. On one hand, technological innovation was identified as an efficient means of curbing CO_2_E, thus exerting a negative impact. On the other hand, the research highlighted the potential for technological innovation to inadvertently contribute to increased energy consumption and subsequent pollution, thereby imparting a positive impact.

### 2.2 Literature review in context of Pakistan

In a series of rigorous studies in Pakistan, Raza and Lin [41] undertook a comprehensive investigation into the potential ramifications of renewable energy substitution and energy technology on the economic development of transitional economies, with a specific focus on Pakistan. Employing a *trans*-log production function as their analytical framework, the researchers revealed compelling evidence indicating that all output and substitution elasticities significantly positively contributed to economic development. Similarly, Lin and Raza [42] conducted an extensive analysis to estimate the prevailing trends in electricity consumption within Pakistan from 1989 to 2018. They identified the economic structure sector as the principal driver behind the country's notable growth in total electricity consumption. Additionally, their investigation unveiled intermittent positive fluctuations in energy intensity. Raza and Lin [43] delved into the intricate factors influencing the levels of CO_2_E arising from electricity generation in Pakistan. Their meticulous examination unveiled the pivotal roles played by the activity effect and population in the escalating CO_2_E. Lin and Raza [44] conducted an in-depth analysis of CO_2_E from Pakistan's power sector from 1978 to 2017. Within this temporal scope, the researchers identified population, economic activity and gross domestic product as pivotal factors driving the observed rise in CO_2_E. Conversely, the effects of carbon intensity and energy intensity were found to play a mitigating role in curbing CO_2_E. Raza and Lin [45] made significant observations by focusing on analyzing energy consumption patterns at the sectorial level in Pakistan. Their investigation revealed a positive energy intensity exhibited by the observed sectors, leading to adverse implications for economic activity. Raza and Lin [46] also found positive impact of renewable energy on the productivity of Pakistan. Ahsan and Iqbal [47] meticulously presented an optimized design to propose viable renewable energy solutions. They assessed the economic feasibility of a grid-tied captive hybrid renewable energy power plant tailored for Pakistan. The researchers' findings provided compelling evidence for the plant's ability to effectively respond to load variations encountered in industrial settings, positioning it as a dependable candidate for meeting designated energy demands. Similarly, Ali et al. [48] comprehensively evaluated hybrid energy systems encompassing solar, wind, battery, and diesel generators. They highlighted that a photovoltaic system coupled with a diesel generator and incorporating a battery storage system emerged as the most feasible option for the selected regions.

### 2.3 Theoretical framework

The circular economy theory is a robust framework to substantiate the positive impact of hybrid energy systems on ES. Rooted in promoting sustainability, this economic model focuses on waste reduction, resource efficiency and material reuse [49]. Hybrid energy solutions, comprising diverse renewable sources to generate electricity, closely align with the principles of the circular economy. By adopting hybrid energy systems, societies can effectively transition away from conventional fossil fuel-based energy generation, which is known to contribute to GHG emissions and environmental degradation [50]. Instead, the integration of renewable energy sources in combination enhances energy production efficiency, diminishes reliance on finite fossil fuels and mitigates carbon emissions, thereby significantly contributing to ES [51].

The circular economy theory emphasizes closing resource loops, minimizing waste and optimizing resource utilization. Hybrid energy systems play a vital role in facilitating the integration of various renewable resources, thereby optimizing their collective use to efficiently meet energy demands [52]. Using and recycling renewable energy sources, the circular economy approach fosters a more sustainable energy system that minimizes environmental impact and supports long-term ecological sustainability [52]. Thus, the circular economy theory provides a comprehensive and coherent foundation for comprehending and endorsing the positive influence of hybrid energy systems on ES. This approach offers a holistic, integrated perspective that harmonizes economic prosperity with ecological preservation.

### 2.4 Hypothesis

Based on existing literature and circular economy theory, the study concludes the positive role of hybrid energy systems in reducing global carbon emissions. The study, therefore, develops the following hypothesis:

H_1_: Hybrid energy systems have significant contributions to environmental sustainability.

### 2.5 Knowledge gap

The existing literature offers valuable insights into the influence of renewable hybrid energy systems on ES, particularly emphasizing their integration within the circular economy framework in Pakistan. Extensive research has explored the positive contributions of diverse energy sources, including SPV, WND, HYDE and NPG, toward enhancing ES. Notably, the significance of hybrid energy systems in advancing ecological sustainability is underscored. However, despite the wealth of studies examining renewable hybrid systems and their impact on environmental quality in various contexts, the specific attention devoted to 5 hybrid systems (presented in this study) and their potential role in mitigating climate change effects in Pakistan still needs to be explored. As such, the research landscape exhibits a notable knowledge gap concerning exploring hybrid energy systems and their potential benefits in promoting environmental protection and climate change mitigation within the unique context of Pakistan.

### 2.6 Conceptual framework

**Fig. 1:**
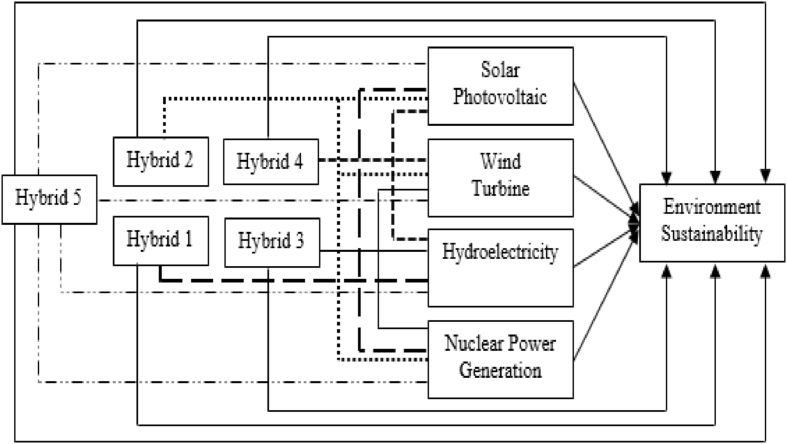
Conceptual framework.

Fig. 1 depicts a comprehensive conceptual framework illustrating the composition of various hybrid energy systems and their respective contributions to environmental sustainability. The framework highlights five distinct hybrid systems, each characterized by a unique combination of renewable energy sources. Hybrid system 1 comprises solar photovoltaic, hydroelectricity and nuclear power generation, while hybrid system 2 integrates solar photovoltaic, wind turbine and nuclear power generation. Likewise, hybrid system 3 encompasses wind turbine, hydroelectricity and nuclear power generation, and hybrid system 4 consists of solar photovoltaic, wind turbine and hydroelectricity. Hybrid system 5 is an all-encompassing combination of solar photovoltaic, wind turbine, hydroelectricity and nuclear power generation. Each hybrid system is visually represented by distinct line styles, emphasizing their characteristics and contributions to environmental sustainability. Moreover, the conceptual framework underscores the significance of each renewable energy source (solar photovoltaic, wind turbine, hydroelectricity and nuclear power generation) and the collective impact of each hybrid system (hybrid 1–5) on environmental protection.

## 3 Methodology

### 3.1 Data and sources

The present study aims to analyze the role of renewable hybrid energy systems of energy on ES using ES as the outcome variable, while SPV, WND, HYDE, NPG, and 5-Hybrid energy methods as explanatory variables. For this purpose, the study uses quarterly data of Pakistan for 2011Q1-2020Q4. The data are gathered from the global economy, with a sample size of 40 observations (10 years × 4 quarters). Although, it is evident from the prior studies that 40 observations are sufficient to attain the statistical power in time series analysis. Several preceding studies have utilized even less than 40 observations in the context of different countries. For concern, Ahmed et al. [53] used data from Pakistan from 1996 through 2018 (22 observations) for predicting environmental degradation. Salahuddin and Gow [54] conducted a South African study using data from 1994 to 2014 (21 observations). Similarly, Erdoğdu and Çiçek [55] utilized Turkish data from 1994 to 2014 (21 observations).

The selection of the time from 2011Q1 to 2020Q4 is strategic and well-justified, as it enables a robust and in-depth analysis of the impact of hybrid energy systems on ES in Pakistan. By encompassing ten years, this period allows for a comprehensive assessment of the long-term effects and trends associated with the adoption and effectiveness of hybrid energy sources. Moreover, it covers a significant portion of the 2010s, a decade marked by notable advancements in renewable energy technologies and substantial global energy policy shifts. Data availability throughout this timeframe ensures the stability and consistency required to examine quarterly trends and variations in relevant variables thoroughly. Consequently, the study can draw sound and evidence-based conclusions regarding the distinct contributions of different hybrid energy systems toward enhancing ecological sustainability.

### 3.2 Variables and construction of hybrid systems

Environmental sustainability (ES): The study measures ES by per capita carbon emissions [53]. Hybrid systems: The study designs five hybrid systems of energy (namely, hybrid 1, hybrid 2, hybrid 3, hybrid 4, and hybrid 5) by taking the averages of different combinations comprising solar photovoltaic, wind, hydroelectricity, and nuclear power generation systems followed by Refs. [[56], [57], [58]]. The combinations of hybrid systems of energy are constructed as SPV-HYDE-NPG (Hybrid 1), SPV-WND-NPG (Hybrid 2), WND-HYDE-NPG (Hybrid 3), SPV-WND-HYDE (Hybrid 4), and SPV-WND-HYDE-NPG (Hybrid 5). Moreover, all the variables were transformed into logarithm natural to avoid the distributive properties issues of data series [59].

### 3.3 Econometric techniques

Initially, the study analyzes the individual impact of SPV, WND, HYDE, and NPG on ES (Equation (1)). Henceforth, the effect of different hybrid systems of energy on ES is analyzed using Equation (2). In this regard, the study formulates the following econometric models:

(1) Model 1: CO_2_E_t_ = β_0_ + β_1_ (SPV_t_) + β_2_ (WIND_t_) + β_3_ (HYDE_t_) + β_4_ (NPG_t_) + e −

(2) Model 2: CO_2_E_t_ = β_0_ + β_1_ (SPV-HYDE-NPG_t_) + β_2_ (SPV-WND-NPG_t_) + β_3_ (WND-HYDE-NPG_t_) + β_4_ (SPV-WND-HYDE_t_) + β_5_ (SPV-WND-HYDE-NPGt) + e −

Where; “CO_2_E is CO_2_ emission, WND: wind, SPV: solar photovoltaic, HYDE: hydroelectricity, NPG: nuclear power generation, β_1_ ----- β_5_: coefficients, β0: constant, e: residuals and *t* denote time."

The study adopts autoregressive distributive lag model (ARDL) to estimate the results. Adopting the ARDL as the analytical approach for this study is methodologically justified. ARDL is a robust econometric technique known for its ability to examine both short-term and long-term relationships between variables, rendering it suitable for analyzing the impact of hybrid energy systems on ES over the specified ten-year period. Additionally, ARDL demonstrates resilience in handling small sample sizes, a crucial factor given the availability of quarterly data for Pakistan. Through the application of ARDL, the study can effectively estimate the effects of distinct hybrid energy sources, including SPV, WND, HYDE and NPG, on ES. As a result, the research aims to yield valuable insights into each hybrid system's contributions to advancing Pakistan's ecological sustainability.

Estimating CO_2_E is crucial in identifying the primary drivers of climate change and facilitating the transition towards renewable and low-carbon energy sources. This assessment is paramount for devising impactful environmental policies to mitigate pollution and advance sustainable development. Accurate CO_2_E estimation contributes significantly to the overarching goal of environmental preservation and climate resilience by addressing the adverse effects of global warming on the environment and human health.

Before ARDL, some precautionary tests (multicollinearity, serial correlation, and heteroscedasticity) are employed. As the present study is time series, the examination of stationarity is a prerequisite. Thus, the study applies ADF and PP tests to detect the problem of stationary. After determining the order of integration, testing whether the long-run relationship is present among the variables is essential. Considering the stationarity properties of the data, the study applies the ARDL bounds testing approach to test the long-run relationship. After choosing the sequence of integration, it is critical to determine whether a long-term relationship exists between the variables. The study examines the long-run relationship using the ARDL limits testing method while considering the data's stationarity properties. ARDL bounds testing approach uses the following unrestricted error correction model to test the co-integrating relation among the selected variables (Equations (3) and (4)).

(3) $${{\left({\Delta CO}_{2}E\right)}_{t}=\partial_{0}+\partial_{1}{\left(SPV\right)}_{t-i}+\partial }_{2}{\left(WND\right)}_{t-i}+\partial_{3}{\left(HYDE\right)}_{t-i}+\partial_{4}{\left(NPG\right)}_{t-i}+\sum_{i=1}^{q}\propto_{1}{\left(\Delta SPV\right)}_{t-i}+\sum_{i=1}^{q}\propto_{2}{\left(\Delta WND\right)}_{t-i}+\sum_{i=1}^{q}\propto_{3}{\left(\Delta HYDE\right)}_{t-i}+\sum_{i=1}^{q}\propto_{4}{\left(\Delta NPG\right)}_{t-i}+\epsilon_{it}$$

(4) $${\left({CO}_{2}E\right)}_{t}=\delta_{0}+\delta_{1}{\left(SPV-HYDE-NPG\right)}_{t-i}+\delta_{2}{\left(SPV-WND-NPG\right)}_{t-i}+\delta_{3}{\left(WND-HYDE-NPG\right)}_{t-i}+\delta_{4}{\left(\text{SPV}-\text{WND}-\text{HYDE}\right)}_{t-i}+\delta_{5}{\left(\text{SPV}-\text{WND}-\text{HYDE}-\text{NPG}\right)}_{t-i}+\sum_{i=1}^{q}\beta_{1}{\left(\Delta SPV-HYDE-NPG\right)}_{t-i}+\sum_{i=1}^{q}\beta_{2}{\left(\Delta SPV-WND-NPG\right)}_{t-i}+\sum_{i=1}^{q}\beta_{3}{\left(\Delta \text{WND}-\text{HYDE}-\text{NPG}\right)}_{t-i}+\sum_{i=1}^{q}\beta_{4}{\left(\Delta \text{SPV}-\text{WND}-\text{HYDE}\right)}_{i,t-i}+\sum_{i=1}^{q}\beta_{5}{\left(\Delta \text{SPV}-\text{WND}-\text{HYDE}-\text{NPG}\right)}_{t-i}+u_{it}$$

Where: Δ is the difference operator, SPV is solar-photovoltaic, WND is wind, HYDE is hydroelectricity, NPG is nuclear power generation, SPV-BTRY-NPG is hybrid 1, PV-WND-NPG is hybrid 2, WND-BTRY-NPG is hybrid 3, SPV-WND-HYDE is hybrid 4, PV-WND-HYDE-NPG is hybrid 5, ∂s and ∝s are variables that are examined to check the long-run relations among variables of equation (3), while δs and βs are variables that are looked for checking long-run association among the variables of Equations (3) and (4).

Equations (3) and (4) use “the F test to examine the co-integrating relation among the study variables. This test involves the testing of the null hypothesis of no co-integration by assuming a zero joint restriction on the ∂s and δs in the error correction (ECM) model (i.e., H_0_: ∂_1_ = ∂_2_ = ∂_3_ = ∂_4_ = 0; and H_0_: δ_1_ = δ_2_ = δ_3_ = δ_4_ = δ_5_ = 0 for equations (3) and (4) respectively). The asymptotic distributions of the test statistics are non-standard irrespective of whether the variables are integrated of order 0, i.e., I (0), or integrated of order 1, i.e., I (1).

After examining the long-run relationship, the study applies the error correction model (ECM) to compute the short-run estimates (Equations (5) and (6)).

(5) $${\left({\Delta CO}_{2}E\right)}_{t}=\partial_{0}+\sum_{i=1}^{q}\propto_{1}{\left(\Delta SPV\right)}_{t-i}+\sum_{i=1}^{q}\propto_{2}{\left(\Delta WND\right)}_{t-i}+\sum_{i=1}^{q}\propto_{3}{\left(\Delta HYDE\right)}_{t-i}+\sum_{i=1}^{q}\propto_{4}{\left(\Delta NPG\right)}_{t-i}+\varphi \;{\left(ECT\right)}_{t-i}\;\epsilon_{t}$$

(6) $${\left({CO}_{2}E\right)}_{t}=\delta_{0}+\sum_{i=1}^{q}\beta_{1}{\left(\Delta SPV-HYDE-NPG\right)}_{t-i}+\sum_{i=1}^{q}\beta_{2}{\left(\Delta SPVWND-NPG\right)}_{t-i}+\sum_{i=1}^{q}\beta_{3}{\left(\Delta \text{WND}-\text{HYDE}-\text{NPG}\right)}_{t-i}+\sum_{i=1}^{q}\beta_{4}{\left(\Delta \text{SPV}-\text{WND}-\text{HYDE}\right)}_{i,t-i}+\sum_{i=1}^{q}\beta_{5}{\left(\Delta \text{SPV}-\text{WND}-\text{HYDE}-\text{NPG}\right)}_{t-i}+\emptyset {\left(ECT\right)}_{t-i}+u_{t}$$

Where; “Δ is the first difference operator, ∂_0_ and δ_0_ are the intercept terms for the case of equations (5) and (6), ∝s and βs are the slope coefficients of short-run for equations (5) and (6), ECT_t-i_ is the error correction term, which signifies the speed of adjustment or the level of long-run equilibrium, and ϵ_t_ and u_t_ is the stochastic error terms.”

## 4 Results

### 4.1 Descriptive statistics

Table 1 provides descriptive statistics for the variables of interest. The LNCO2E variable exhibits a mean of −0.0512 and a median of −0.0618, indicating an average LNCO2E of −5.12% for Pakistan from 2011 to 2021. The lower median relative to the mean suggests a right-skewed distribution, implying a tendency for LNCO2E to be skewed towards higher values for most years. The standard deviation (Std. Dev.) of 0.0329 indicates notable variation in LNCO2E across the years. Similarly, for the HYDE variable, the mean and median are 3.4430 and 3.4515, respectively, signifying an average HYDE of 3.44% during the same period. The right-skewed distribution of HYDE, supported by its higher median value, is evident from the Std. Dev. of 0.0797, denoting substantial temporal variability. Additional descriptive measures for the remaining variables are also presented in Table 1. Moreover, the minimum and maximum values of the variables fall within the range of −4.6051 to 3.3196 and −0.0105 to 3.5734, respectively. It is worth noting that data exhibiting skewness and kurtosis values between −2 and +2 and −7 and +7, respectively, are considered normal distributions. Table 4.1 confirms that both skewness and kurtosis values satisfy these criteria, affirming the normal distribution of the data.

**Table 1:** Descriptive statistics.

Particulars	HYDE	LNCO_2_E	NPG	SPV	WND
**Mean**	3.4430	−0.0512	1.6035	−1.6159	−1.2857
**Median**	3.4515	−0.0618	1.5216	−1.4271	−0.2357
**Maximum**	3.5734	−0.0105	2.2289	0.4382	0.7419
**Minimum**	3.3196	−0.0943	0.9400	−4.6051	−3.0062
**Std. Dev.**	0.0797	0.0329	0.3687	1.6557	2.0310
**Skewness**	−0.029	0.1763	0.1119	−0.4067	−0.5843
**Kurtosis**	2.0787	1.5886	2.6026	2.0156	1.6350
**Probability**	0.5277	0.2044	0.8555	0.2943	0.1887

### 4.2 Test of stationarity

The results of the ADF and PP unit root tests used to examine the stationarity characteristics and sequence of integration are presented in Table 2. Two cases are dealt with using both tests (with intercept and with trend and intercept). By rejecting the null hypothesis of non-stationary series at the 10%, 5%, and 1% level of significance, the results in Table 2 imply that the chosen variables are integrated in the mix order; some variables are stationary at a level, while others are stationary at the first difference.

**Table 2:** Test of stationarity.

Variables	Augmented Dickey-Fuller: ADF				Decision
	Level		First difference		
	Trend	Trend & Intercept	Trend	Trend & Intercept	
**SPV**	1.6734	0.8735	−3.3566^b^	−4.3465^a^	I (1)
**WND**	−3.8263^b^	−2.2821^b^	−4.3645^a^	−4.9485^a^	I (0), I (1)
**HYDE**	0.8373	1.8745	−4.8857^a^	−5.7027^a^	I (1)
**NPG**	−2.9254^c^	−3.0067^b^	−5.0456^a^	−6.6344^a^	I (0), I (1)
**Hybrid 1**	−8.9144^a^	−7.6344^a^	−8.3764^a^	−8.0003^a^	I (0), I (1)
**Hybrid 2**	1.9372	0.2763	−3.6455^b^	−3.9074^a^	I (1)
**Hybrid 3**	2.3327	2.6087	−3.9964^b^	−4.8734^a^	I (1)
**Hybrid 4**	−4.9780^a^	−4.0037^b^	−5.9833^a^	−5.9873^a^	I (0), I (1)
**Hybrid 5**	−2.2376^c^	−3.3745^b^	−4.2982^a^	−5.3875^a^	I (0), I (1)
**CO**_**2**_**E**	1.13563	1.19036	−2.8345^b^	−3.2634^a^	I(1)
Variables	**Phillips*–*Perron: PP**				**Decision**
	**Level**		**First difference**		
	**Trend**	**Trend & Intercept**	**Trend**	**Trend & Intercept**	
**SPV**	1.5342	0.1534	−3.169^a^	−3.3874^a^	I (1)
**WND**	−2.1563^c^	−2.9664^c^	−3.0254b	−4.9845^a^	I (0), I (1)
**HYDE**	1.5645	1.7753	−3.7856^b^	−5.8735^a^	I (1)
**NPG**	−2.9984^c^	−3.1764^b^	−3.9746^a^	−4.8745^a^	I (0), I (1)
**Hybrid 1**	−4.8724^a^	−5.8353^a^	−5.3344^a^	−6.3373^a^	I (0), I (1)
**Hybrid 2**	2.0987	2.3017	−3.6291^a^	−3.8234^a^	I (1)
**Hybrid 3**	−3.8723^b^	−4.1654^a^	−4.7005^a^	−4.9656^a^	I(0), I (1)
**Hybrid 4**	−5.7835^a^	−6.2437^b^	−6.0024^a^	−6.9826^a^	I (0), I (1)
**Hybrid 5**	−2.9936^c^	−3.0263^b^	−3.9746^a^	−4.7624^a^	I (0), I (1)
**CO**_**2**_**E**	1.0036	1.1193	−4.3874^a^	−5.0026^a^	I(1)

Note: a: p ≤ 0.01; b: p ≤ 0.05; c: p ≤ 0.10.

### 4.3 Diagnostic tests

To ensure the reliability of the econometric analysis, this study conducted various diagnostic tests to identify potential issues such as multicollinearity, serial correlation, heteroscedasticity (HSK) and model stability. The correlation matrix, Breusch-Godfrey serial correlation test and Breusch-Pagan-Godfrey HSK test were employed for detecting multicollinearity, serial correlation and HSK, respectively. Moreover, the Ramsey Reset test was utilized to assess the robustness of the econometric models. The results of these diagnostic tests are presented in Table 3. The findings in panel A of Table 3 indicate that the data are free from multicollinearity problems, as the correlation coefficients between variables are all below 0.5, signifying no significant correlation issues among the independent variables. Panels B and C report insignificant test statistics, suggesting the absence of serial correlation and HSK problems, thereby assuring the data's reliability. Furthermore, the non-significant value obtained from the Ramsey Reset test indicates that the econometric models accurately represent the data and any potential econometric errors do not adversely affect the analysis. These comprehensive diagnostic tests strengthen the validity and robustness of the study's econometric analysis, enhancing confidence in the obtained results.

**Table 3:** Diagnostic tests.

Panel A: Multicollinearity					
	**HYDE**	**NPG**	**SPV**	**WND**	**CO**_**2**_**E**
**HYDE**	**1**				
**NPG**	0.1675	**1**			
**SPV**	0.2611	0.1291	**1**		
**WND**	0.2635	0.2827	0.275	**1**	
**CO**_**2**_**E**	0.1771	0.6695	0.1766	0.2611	**1**
Panel B: Serial Correlation LM Test					
**Breusch-Godfrey**	**Model 1**		**Model 2**		**Decision**
	***Test statistic***	***Probability***	***Test statistic***	***Probability***	*No serial correlation*
	1.3744	0.1253	0.2734	0.1894	
Panel C: Heteroscedasticity					
**Breusch-Pagan-Godfrey**	**Model 1**		**Model 2**		**Decision**
	***Test statistic***	***Probability***	***Test statistic***	***Probability***	*No heteroscedasticity*
	1.6734	0.1184	1.0264	0.1174	
Panel D: Model Stability					
**Ramsey Reset test**	**Model 1**		**Model 2**		**Decision**
	***Test statistic***	***Probability***	***Test statistic***	***Probability***	*Models are correctly specified*
	1.244	0.1999	1.1194	0.1974	

### 4.4 ARDL bounds Co-integration test

The ARDL bounds test is a crucial tool for determining the presence of a long-run relationship between variables in a co-integrated system. It aids in selecting the appropriate ARDL model based on the order of integration. The critical bound values presented in Table 4 are pivotal in evaluating the statistical significance of the F-statistic derived from the bounds test. However, certain assumptions must be met to ensure the validity and reliability of the results. Firstly, all variables involved in the analysis must exhibit stationarity or co-integration of the same order. Secondly, the model must be free from endogeneity concerns, implying that the independent variables remain unaffected by the error term. Thirdly, the bounds test assumes the absence of spurious regression, whereby non-stationary variables do not demonstrate a significant relationship by chance. Adherence to these fundamental assumptions is paramount to deriving meaningful and robust outcomes from the bounds test, enabling researchers to draw appropriate conclusions regarding long-run relationships among the variables under study. The output is reported in Table 4 which shows that the value of the F statistic (3.8564 and 4.1094) is greater than the upper bound at the significance level of 0.10, 0.05, and 0.01% for models 1 and 2, respectively. Thus, it is concluded that all variables move together in the long run.

**Table 4:** ARDL Bounds Test and its Critical Bound Values Range.

F statistic	Model 1		Model 2	
	F-Statistic	K	F-statistic	K
	3.8564	4	4.1094	5
Critical bounds	**I**_**0**_**bound**	**I**_**1**_**bound**	**I**_**0**_**bound**	**I**_**1**_**bound**
10%	1.99	2.94	1.99	2.94
5%	2.27	3.28	2.27	3.28
1%	2.55	3.61	2.55	3.61

### 4.5 ARDL regression

#### 4.5.1 Effects of short run relationships between parameters

Short-run results for the impact of hybrid energy systems are reported in Table 5. The ECM value of −0.6754 (−0.6993) in model 1 (model 2) indicates that 67.54% (69.93%) inconsistency between long and short-term ES can be corrected within a year.

**Table 5:** **Results:** Short Run/ECM.

DV: CO_2_E	Model 1		Model 2	
Independent Variables	Coefficient	Probability	Coefficient	Probability
CO_2_E(-1)	0.1914	0.0000^a^	0.2864	0.0000^a^
HYDE	−0.0018	0.0182^a^	–	–
HYDE(-1)	0.0015	0.0431^b^	–	–
NPG	−0.0159	0.0000^a^	–	–
NPG(-1)	0.0096	0.0102^a^	–	–
SPV	0.1286	0.0000^a^	–	–
SPV(-1)	−0.0907	0.0028^a^	–	–
WND	−0.0225	0.1006	–	–
WND(-1)	0.0212	0.1256	–	–
HYBRID1	–	–	2.9419	0.0000^a^
HYBRID1(-1)	–	–	−1.8775	0.0169^a^
HYBRID2	–	–	0.8693	0.0000^a^
HYBRID2(-1)	–	–	−0.6683	0.0035^a^
HYBRID3	–	–	1.2846	0.0207^b^
HYBRID3(-1)	–	–	−1.1635	0.0325^b^
HYBRID4	–	–	0.0726	0.0055^a^
HYBRID4(-1)	–	–	−0.0671	0.0128^a^
HYBRID5	–	–	−5.2647	0.0000^a^
HYBRID5(-1)	–	–	3.8513	0.0041^a^
ECM	−0.6754	0.0000^a^	−0.6993	0.0000^a^
C	0.01634	0.0034^a^	0.07254	0.0368^b^

Note: a: p ≤ 0.01; b: p ≤ 0.05.

#### 4.5.2 Effects of long run relationships between parameters

The study applies the ARDL bounds integration approach to test the long-run association between the variables. Results of model 1 (as reported in Table 6) show significant contributions of SPV, WND, and NPG in CO_2_E. A negative sign with the coefficients depicts that a 1-unit increase in SPV, WND, and NPG tends to reduce 0.0229, 0.0042, and 0.0204 units of CO_2_E at 5%, 1%, and 5%, respectively. The result implies that SPV has higher contributions in reducing CO_2_E or protecting the environment as the SPV coefficient is high compared to WND, HYDE, and NPG. In comparison, NPG contributes less to preserving the environment than SPV, as depicted by its coefficient of 0.0204. The electricity generation through WND contributes less to improving environmental quality, followed by NPG and SPV, as it has a smaller coefficient (−0.0042). Moreover, electricity production through HYDE does not significantly contribute to ES. The results explain that SPV, NPG, and HYDE contribute 2.29%, 2.04%, and 0.42% to protecting the environment. Output in model 2 of Table 6 shows that hybrid systems of energy (Hybrid 1,2,3,4 and 5) exert a positive (negative) impact on ES (CO_2_E), but the intensity of each hybrid system is different.

**Table 6:** Results: Long-run.

DV: CO_2_E	Model 1		Model 2	
Independent Variables	Coefficient	Probability	Coefficient	Probability
SPV	−0.0229^b^	0.0340	–	–
WND	−0.0042^a^	0.0004	–	–
HYDE	−0.0139	0.1124	–	–
NPG	−0.0204^b^	0.0320	–	–
HYBRID1	–	–	−0.1436^b^	0.0317
HYBRID2	–	–	−0.1212^c^	0.0745
HYBRID3	–	–	−0.2219^a^	0.0093
HYBRID4	–	–	−0.1767^b^	0.0475
HYBRID5	–	–	−0.2680^a^	0.0001
R^2^	0.7146		0.7354	
Adj. R^2^	0.6564		0.6765	

Note: Hybrid 1: SPV-HYDE-NPG, Hybrid 2: SPV-WND-NPG, Hybrid 3: WND-HYDE-NPG, Hybrid 4: SPV-WND-HYDE, Hybrid5: SPV-WND-HYDE-NPG; Note: a: p ≤ 0.01; b: p ≤ 0.05; c: p ≤ 0.10.

The coefficient of each hybrid system depicts that hybrid 5, hybrid 3, hybrid 4, hybrid 1, and hybrid 2 have 2.68%, 2.21%, 1.76%, 1.43%, and 1.21% contribution to ES. Hybrid 1 has a higher contribution to ecological sustainability as compared to hybrid 2. In comparison, hybrid 4 has a more significant contribution in reducing the CO_2_E compared to hybrids 1 and 2, and hybrid 3 has a more substantial contribution in improving the environmental quality compared to hybrids 1, 2, and 4. While hybrid 5 is dominant on all four hybrid systems in reducing the CO_2_E. Fortunately, and as expected, the hybrid 5 energy system is more environmentally friendly and has the highest contribution to ES. The above outcomes support hypothesis 1. Furthermore, the R^2^ of Model 1 and Model 2 are 71.46% and 73.54%, respectively, showing that the models fit well.

### 4.6 Model stability

**Fig. 2:**
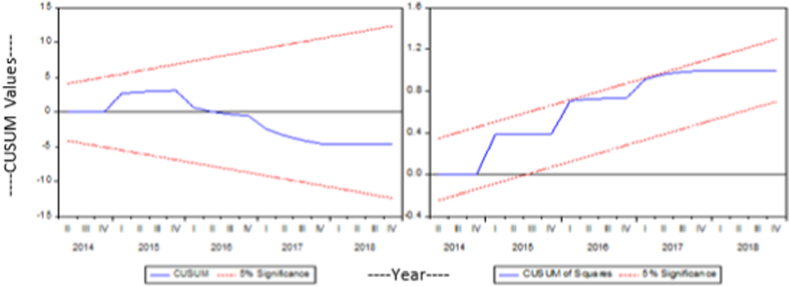
Cusum and Cusum square of model 1.

**Fig. 3:**
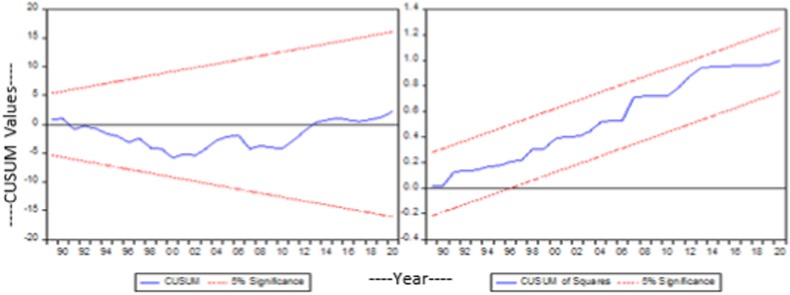
Cusum and Cusum square of model 2.

The CUSUM and CUSUM of the square tests are used in this study to determine whether models are stable [60]. Fig. 2, Fig. 3 display the outcomes. As all of the plots remain inside the crucial bounds, the results show that the current investigation models are stable and accurately stated.

## 5 Discussion

The study analyzes the impact of hybrid energy systems on ES by reducing the effects of climate change and found that electricity generation through SPV, WND, HYDE, and NPG plays a positive role in protecting the environment. Hybrid systems of energy (Hybrid 1,2,3,4 and 5) also positively impact ES. Among five hybrid systems, hybrid 5 (SPV-WND-HYDE-NPG) the energy system is more environmentally friendly and contributes most to ecological sustainability. Our findings align with prior studies [[21], [22], [23], [24], [25],[28], [29], [30], [31], [32], [33], [34], [35], [36], [37],[41], [42], [43], [44], [45], [46], [47], [48]] and circular economy theory. Thus, H1 is accepted.

The study's findings present compelling evidence for the positive impact of integrating renewable energy sources on ES in Pakistan. The individual contributions of SPV, NPG and HYDE, with 2.29%, 2.04%, and 0.42%, respectively, underscore their significance in promoting a greener and more sustainable energy landscape within the country. Additionally, the study highlights the importance of adopting hybrid energy systems, including Hybrid 1, 2, 3, 4, and 5, to further enhance ES.

Notably, the study identifies the hybrid-5 energy system as the most environmentally friendly option, with the highest contribution to ES. This finding is particularly relevant to Pakistan, where addressing ecological challenges like climate change and air pollution is crucial for the country's development and public health [61,62]. As Pakistan aims to intensify efforts to combat climate change and protect the environment, the insights from this research assume paramount importance. Integrating SPV, WND, HYDE and NPG in hybrid energy systems can effectively mitigate the negative impacts of conventional energy sources, reduce carbon emissions and foster long-term ecological well-being within the nation.

These findings have significant implications for policymakers in Pakistan, urging them to prioritize adopting effective policies and measures that accelerate the deployment of renewable hybrid energy systems. Embracing such sustainable energy solutions aligns with Pakistan's commitment to international sustainability goals, promotes a low-carbon economy and contributes to a more environmentally responsible energy sector. By implementing these recommendations, Pakistan can pave the way toward a more sustainable and resilient energy infrastructure, ensuring a cleaner and healthier environment for the well-being of its current and future generations [63,64].

## 6 Conclusion and policy implications

### 6.1 Conclusion

This study provides robust evidence supporting the pivotal role of hybrid energy systems in advancing ES within the context of the circular economy network. The thorough analysis establishes the positive contributions of SPV, WND, HYDE and NPG to enhancing ES. The implementation of diverse hybrid energy systems demonstrates their favorable impact on ES, notably with the hybrid-5 system emerging as the most environmentally friendly and significantly contributing to ES. Given the compelling findings, it becomes imperative for policymakers and officials of the Pakistani government to prioritize and actively promote the integration of hybrid energy systems into the national energy landscape. Embracing such systems provides a promising pathway toward the circular economy and fosters the long-term preservation of ecological balance. By embracing renewable energy sources like SPV, WND, HYDE and NPG in hybrid configurations, Pakistan can effectively combat climate change, reduce carbon emissions and foster sustainable economic development. These initiatives hold the potential to lead the nation towards a cleaner, greener and more resilient future, aligning with global endeavors to address pressing environmental challenges. The valuable insights from this study offer essential guidance in shaping effective policies and strategies that will facilitate the widespread adoption of hybrid energy systems, reinforcing Pakistan's commitment to a sustainable and prosperous future.

### 6.2 Policy implications

Over the past decade, Pakistan has demonstrated commendable progress in embracing renewable energy sources and fostering sustainable development. The country has proactively deployed renewable energy projects, particularly emphasizing solar and wind power generation. Government-led initiatives offering incentives for private-sector investment in renewable energy have stimulated the establishment of numerous solar and wind farms nationwide. Additionally, Pakistan has shown a strong commitment to the development of hydropower projects, capitalizing on its abundant water resources. The study's findings align harmoniously with these national endeavors, emphasizing the favorable impact of renewable energy sources, including SPV, WND, HYDE and NPG, on enhancing ES. Leveraging the potential of these sources within hybrid energy systems holds the promise of further augmenting ES and curbing carbon emissions. As Pakistan continues to address its energy and environmental challenges, the study's results provide a vital guiding framework to strengthen and expand existing sustainable energy initiatives, solidifying the country's path toward a cleaner and greener future.

The study's results have substantial practical implications for advancing ES in Pakistan. The findings underscore the favorable influence of integrating renewable energy sources within the national energy portfolio. Notably, SPV, NPG and HYDE contribute positively to ES, highlighting the significance of prioritizing investments in these renewable technologies to reduce carbon emissions and mitigate harmful pollutants effectively. Moreover, adopting hybrid energy systems, particularly hybrid-5 (SPV-WND-HYDE-NPG), is a pragmatic approach to bolster ecological sustainability. Policymakers and relevant stakeholders can leverage these research insights to formulate and implement effective energy policies, facilitating a seamless transition towards cleaner and more sustainable energy alternatives. Promoting renewable hybrid energy infrastructure elevates environmental quality, ensures energy security, minimizes fossil fuel dependency and fosters long-term socio-economic benefits, consolidating Pakistan's commitment to sustainable development.

The study's outcomes hold particular significance for various officials, including policymakers, government authorities and energy regulatory bodies in Pakistan. As principal drivers of the country's energy policies, these officials can draw upon the research findings to design strategic initiatives prioritizing the seamless integration of renewable energy sources, such as SPV, WND, HYDE and NPG, into the national energy landscape. By incorporating these clean energy solutions, Pakistan can chart a trajectory towards a more sustainable and environmentally conscious energy sector. Furthermore, environmental agencies and sustainability-focused organizations can leverage the study's insights to advocate for and actively support the widespread adoption of cleaner and greener energy options. Aligning policies and initiatives with the study's recommendations can foster collective efforts in combatting climate change, curbing pollution and advancing the nation's pursuit of a healthier and sustainable future.

### 6.3 Limitations and future recommendations

Despite providing valuable insights into the positive impact of hybrid energy systems on ES, this study has several noteworthy limitations with future recommendations. Firstly, the focus on data exclusively from Pakistan raises concerns about the generalizability of the results to other regions with distinct energy compositions and environmental challenges. Secondly, relying on quarterly data may only partially capture the long-term trends and dynamics in the intricate relationship between energy sources and the environment. Furthermore, using the ARDL assumes linear relationships, potentially oversimplifying the complex interactions among energy sources and sustainability indicators. To address these limitations, future research endeavors could conduct cross-country analyses to comprehensively assess the effectiveness of hybrid energy systems across diverse contexts. Longitudinal studies with extended time frames offer a more comprehensive understanding of the enduring impact of hybrid systems on ES. Additionally, embracing more sophisticated econometric techniques like nonlinear modeling could provide deeper insights into the complex energy-environment relationship. Furthermore, future research could investigate the socio-economic implications of adopting hybrid energy systems and identify pertinent policy implications to facilitate a seamless transition towards sustainable energy practices.

## Funding statement

This work was supported by Liaoning Revitalization Talents Program (grant number: XLYC2002116).

## Data availability statement

Data associated with this study has been deposited at https://www.theglobaleconomy.com/, https://databank.worldbank.org/source/world-development-indicators.

## Declaration of competing interest

The authors declare that they have no known competing financial interests or personal relationships that could have appeared to influence the work reported in this paper.
